# Variety of rumen microbial populations involved in biohydrogenation related to individual milk fat percentage of dairy cows

**DOI:** 10.3389/fvets.2023.1106834

**Published:** 2023-03-02

**Authors:** Lei Zhang, Hong Shen, Jiyou Zhang, Shengyong Mao

**Affiliations:** ^1^Ruminant Nutrition and Feed Engineering Technology Research Center, College of Animal Science and Technology, Nanjing Agricultural University, Nanjing, China; ^2^Laboratory of Gastrointestinal Microbiology, Jiangsu Key Laboratory of Gastrointestinal Nutrition and Animal Health, National Center for International Research on Animal Gut Nutrition, College of Animal Science and Technology, Nanjing Agricultural University, Nanjing, China; ^3^Bioinformatics Center, Academy for Advanced Interdisciplinary Studies, Nanjing Agricultural University, Nanjing, China

**Keywords:** milk fat percentage, rumen microbiome, biohydrogenation, linoleic acid isomerase, dairy cows

## Abstract

Our objective was to investigate the contribution of the rumen microbiome on the individual milk fat percentage (MFP) of Holstein dairy cows under the same nutritional and management conditions. From 92 early lactation dairy cows, the top 10 with the highest MFP (HF; *n* = 10) and the last 10 with the lowest MFP (LF; *n* = 10) were selected for the study. As a result, the milk *trans-*10, *cis-*12 C18:2 content was significant lower in the HF group than that in the LF group (*P* < 0.001). The rumen acetate to propionate ratio was significant higher in the HF group than that in the LF group (*P* = 0.035). According to the results of 16S rRNA gene sequencing, a minor but significant difference existed between the groups (*P* = 0.040). Three genera of the family Lachnospiraceae and four genera of the order Bacteroidales were identified to be the biomarkers for the LF group and HF group in the LEfSe analysis, respectively. Three microbial modules enriched by the family Lachnospiraceae were positively related to the milk *trans-*10, *cis-*12 C18:2 content (*r*_*s*_ > 0.60, *P* < 0.05). According to the results of shotgun metagenome sequencing, three kinds of linoleic acid (LA) isomerase genes were present in the gene pools of the rumen microbiome. Among them, the relative abundance of *Bifidobacterium* LA isomerase (BBI) was higher in the HF group than that in the LF group (*P* = 0.007). Three metagenome-assembled genomes (MAGs) with LA isomerase genes were positively correlated to the milk *trans-*10, *cis-*12 C18:2 content (*r*_*s*_> 0.40, *P* < 0.05). Furthermore, all of these three MAGs were found to be able to produce lactate. Taken together, these results indicate that the increased relative abundance of microbial population with the *trans*-10 biohydrogenation pathway within the rumen microbiome contributes to the decrease of MFP *via* the increase of rumen *trans-*10, *cis-*12 C18:2 production. This study provides a new perspective for the development of measures for improving the milking performance of dairy cows.

## 1. Introduction

Milk fat percentage (MFP) is an important indicator of the milk quality and performance of dairy cows. Compared with other nutritional components of the milk, MFP vary to a greater extent (1). Many factors have been found to affect the MFP, such as the breed, diet formula, lactation period, and feeding system (2–4). Even under the same diet and feeding system, the MFP of Holstein dairy cows can vary between 3.0 and 5.0% (5). Recent studies have shown that the individual differences in the composition and metabolic gene profile of the rumen microbiome contribute to variances in MFP (5, 6). However, the underlying roles of rumen microbiome in the modulation of MFP are not as yet understood.

Milk fatty acids (MFA) are the basic units of milk fat. MFAs are derived in two ways: (1) short and medium chain FA (SMCFA, C4-C14) and 50% of C16 FA are *de novo* synthesized from mainly acetate by mammary epithelial cells, and (2) long chain fatty acids (LCFA, >C16) and another 50% of C16 FA are absorbed from blood (7). In ruminants, the rumen microbiome is responsible for the fermentation of dietary carbohydrate into acetate and also for the lipolysis of dietary fat into free FA. Accordingly, the supply amounts of the above MFA precursors partly depend on the fermentation and lipolysis efficiency of the rumen microbiome. In addition, dietary fats for ruminants mostly consist of unsaturated FA (UFA), especially linoleic acid (LA; *cis-*9,12 C18:2) and a-linolenic acid (ALA; *cis-*9,12,15 C18:3). As a detoxifying adaptation, the isomerization from a *cis-*to a *trans-*geometric configuration and the reduction of UFA to saturated FA (SFA), i.e., biohydrogenation (BH), is extensively implemented by ruminal microbes (8). Studies have shown that the composition of BH intermediates, such as the contents of *trans-*10, *cis-*12 C18:2 and *trans-*10 C18:1, and the ratio of *trans-*10 to *trans-*11 C18:2, is associated with the variances of MFP (7, 9, 10). Accordingly, rumen BH pathways and levels depend on the compositions and metabolic properties of the BH microbial population and are also considered to be the important factors affecting MFP.

In this study, we investigated the differences in the fermentation performance, species composition, and lipids metabolism process of the rumen microbiome, with special attention being paid to the composition and metabolism of the microbial population involved in BH, in dairy cows with extremely high and low MFP. We used 16S rRNA gene sequencing and shotgun metagenome sequencing methods in order to expand our understanding of the contribution of the rumen microbiome to the individual performance of MFP.

## 2. Materials and methods

### 2.1. Animals and diets

All experimental procedures involving animals were approved by the Animal Care and Use Committee of Nanjing Agricultural University, in compliance with the Regulations for the Care and Use of Animals (Nanjing Agricultural University, 2018). In total, 92 early lactation Holstein cows of the same parity reared at a commercial dairy farm (Shanghai, China) were used in this study, where they were housed in tie stalls and had free access to water. The total mixed ration (TMR) was provided three times per day at 0,300, 1,200, and 1,700 (forge/concentrate, 40:60, Supplementary Table 1). The chemical analysis of feed samples was based on dry matter (DM) content after drying at 105°C for 2 h (AOAC) (11).

The period of sampling was 3 days. The milk-yield-record and sample-collections of the 92 cows were conducted on d 1–3 at 0,200, 1,100, and 1,600 during experiment. The daily milk samples were mixed in 4:3:3 corresponding to 0,200, 1,100, and 1,600 milking time for the measurement of compositions by a spectrophotometer in Bright Dairy & Food Co., Ltd (Shanghai). The power analysis conducted by G^*^Power v.3.1.9.2 revealed that the sample size of five of each group could receive more than 80% power-value. Subsequently, the top 10 cows with the highest MFP (HF group: MFP > 4.11%, Parity = 2, days in milking, DIM = 63 ± 4), and the last 10 cows with the lowest MFP (LF group: MFP <3.38%, Parity = 2, DIM = 59 ± 2) were selected for the following study (Supplementary Figure 1A). The milk samples of HF and LF cows on d 3 were mixed as before and used for the measurement of LCFA profile. The lactating performance of both groups was recorded during the following 5 weeks.

### 2.2. Rumen contents collection and fermentation index measurement

Rumen contents from 20 cows of two groups were collected at 4 h after a morning feed *via* oral stomach tubes on d 3 and were then divided into two portions (12). The first portion used to extract microbial DNA for 16S rRNA gene and metagenomic sequencing were transferred into sterile tubes, which were immediately placed into liquid nitrogen. The second portion was first squeezed through 4-layers-gauze for pH measurement and then stored at −20°C for the measurement of the fermentation index and LCFA profile. The methods of determination of NH_3_-N and volatile fatty acids (VFA) concentrations were described by Weatherburn et al. (13) and Qin et al. (14), respectively.

### 2.3. Milk and rumen FA profile analysis

The extractions of LCFA from milk and rumen fluid were based on fatty acid (FA) methylation (15). Non-adecanoic acid methyl ester (M102326, Aladdin, Shanghai, China) was used as an internal standard. The GC system comprised an Agilent 8,890 instrument fitted with an autosampler (Agilent 7693A) and equipped with a CP-Sil 88 capillary column. The standards for quantification were FA methyl ester (FAME) mixtures (18919-1AMP, Supleco, Germany), Methyl *trans-*11 C18:1 (CDAA-253185M, ANPEL, China) and Methyl *trans-*10, *c*12 C18:2 (CDAA-258061M, ANPEL, China). The initial column oven temperature of 150°C for 5 minutes was increased at 2°C/min to 175°C, which was held for 15 min, followed by an increase at a rate of 7°C/min to 200°C, which was held for 20 min, and a final increase at 5°C/min to 220°C, which was held for 25 min. The carrier gas and fuel gas were nitrogen and hydrogen at a flow rate of 1.1 and 40 mL/min, respectively. Meanwhile, the temperature of the injector and detector were 260 and 280°C, and the split ratio was set at 20:1.

### 2.4. Total DNA extraction

The rumen contents from 20 cows of LF and HF groups were thawed at 4°C, and then, their microbial DNA were extracted using hexadecyltrimethylammonium bromide (16). The concentration and quality of extracted DNA were evaluated by a spectrophotometer and gel electrophoresis, respectively. Subsequently, the extracted DNAs were used in 16S rRNA gene and shotgun metagenomic sequencing.

### 2.5. 16S rRNA gene sequencing and analysis

The 341F/806R primers were used to amplify the V3-V4 region of the bacterial 16S rRNA genes, and the amplicons were sequenced on an Illumina MiSeq PE250 platform (17). The QIIME2 software suite (version: 2021.2) was applied for the later analysis (18). Briefly, raw reads were qualified, denoised, classified, and counted to generate an amplicon sequence variants (ASVs) table *via* the DADA2 pipeline (19). The SILVA database (SSU Ref NR 99, release 138) was used to annotated ASVs (20). After the random rarefication of the ASV counts of all samples to 18,984 (the lowest number), the α-diversity and β-diversity of bacterial communities were calculated based on the plugin *diversity*. Non-parametric permutational multivariate ANOVA (PERMANOVA) was conducted by the *scikit-bio* package *(http://scikit-bio.org/)* in Python. The differentially abundant species with the biological relevance to the groups, referred to as the biomarker, were assigned to those ASVs whose Linear discriminant analysis (LDA) scores were more than 2 or <-2 in the LDA Effect Size (LEfSe) test (21).

The co-occurrence network of ASVs was constructed according to Spearman's correlations analysis, where only significantly positive correlations (Spearman's R > 0.6) were visualized *via* the Fruchterman-Reingold layout with 9,999 permutations by *igraph* in R (22). The significantly different ASVs within the network were calculated by *Deseq2* (23). We used fast-greedy modularity optimization algorithm (24) to identify modules. The correlations of the detected modules and C18 FAs were identified by Spearman's rank correlation analysis.

### 2.6. Shotgun metagenomic sequencing and analysis

The metagenome libraries were sequenced on an Illumina HiSeq X Ten platform (PE150 mode). The quality of raw reads was assessed by Trimmomatic v.0.33 (25). The qualified reads were compared with the NCBI reference genomes of both host (*Bos Tarus*, GCA_002263795.2) and plant-originated components in the diets, which were maize (*Zea mays*, GCA_003185045.1), soybean (*Glycine max*, GCA_000004515.4), medicago (*Medicago truncatula*, GCA_000219495.2), oat (*Avena sativa*, GCA_022788535.1) and cotton (*Gossypium arboretum*, GCA_025698485.2), to eliminate the data contamination by BWA-MEM v.0.07 (26). The contigs were assembled by MEGAHIT v.1.1.1 (27) from the remaining clean reads, and then used to predicted the open reading frames (ORFs) by Prodigal v.2.6.3 (28). Based on these ORFs, a non-redundant gene set was generated by CD-HIT (29) with the criteria of identity more than 95% and overlap more than 90%. The relative abundance was calculated in transcripts per million (TPM) (30).

The available gene sequences of lipase were downloaded from LED databases (31). So far, LA/ALA isomerase has been divided into *Bifidobacterium* isomerase (BBI, produce *trans*-11 isomers), *Lactobacillus* isomerase (LAI, produce *trans*-10 or *trans*-11 isomers) and *Propionibacterium* isomerase (PAI, produce *trans*-10 isomers), based on the species and isomeric production (32). In this study, the available gene sequences of PAI (ADE00997), BBI (PCT/CN2019/1218), and LAI (ADD22720.1; CBY89653.1; QTP12276.1) were downloaded from GenBank database (33), respectively. BLASTp (34) was used to detected the above genes from the non-redundant gene set, with the parameters of E-value <1e-5 and minimum sequence identity of 40%. The sequence of isomerase was first aligned with mafft (35) and then used to construct a phylogeny tree with fasttree2 (36). The differences in the relative abundances of the genes were analyzed by the Wilcoxon rank-sum test. Differences were considered as significance at a false discovery rate (FDR) <0.05.

MetaBAT2 v.2.11.1 was used to assemble metagenomic bins (MAGs) (37). CheckM v.1.0.7 (38) was used to evaluated the completeness and contamination of aseembled MAGs. dRep v.3.0.1 (39) was used to remove the replicated MAGs. The taxonomy of dereplicated MAGs whose completeness more than 50% and contamination <10%, referred to as high-quality MAGs, were assigned by GTDB-Tk v.1.5.0 (40). We used metaWRAP v.1.1.0 (41) to calculate the relative abundance of each MAG, and used PhyloPhlAn v.3.0 (42) to analyze the phylogenetic relationships of the high-quality MAGs. Finally, the genes were predicted from the sequences of MAGs. The phylogeny tree of MAGs which was visualized by *ggtree* package (43) in R.

### 2.7. Statistical analysis

To make sure the statistical efficiency of this study, the differences in the rumen fermentation index, milk FAs, and rumen FAs concentrations between the groups were analyzed by a two-sided Student *t*-test, where *P* < 0.05 was defined as significantly different.

## 3. Results

### 3.1. Lactation performance

The lactation performances of 92 cows are listed in Supplementary Table 2. With regard to the lactation performance of the 20 selected dairy cows, no significant differences (*P* > 0.05), except for those in MFP and milk fat yield (*P* < 0.001), were found in body weight, DIM, milk yield, milk protein percentage, or lactose percentage between the groups (Table 1). Moreover, the differences in MFP remained significant (*P* < 0.001) during the following 5 weeks after sampling (Supplementary Figure 1B).

**Table 1:** The lactation performances of Holstein cows with highest milk fat percentage (HF) or lowest milk fat percentage (LF).

	**HF**	**LF**	**SEM**	***P* value^a^**
Milk fat (%)	4.30	3.28	0.12	<0.001
Milk protein (%)	3.03	2.88	0.04	0.071
Milk lactose (%)	5.28	5.20	0.03	0.122
Milk yield (kg)	34.70	38.80	1.14	0.067
Milk fat yield (kg)	1.50	1.30	0.05	0.016
Days in milking	62.50	58.90	3.17	0.584
Body weight (kg)	634.21	591.92	16.66	0.214

a P values were calculated by student t-test.

### 3.2. Rumen fermentation index and LCFA compositions

Among the rumen fermentation index, the propionate proportion was significant lower (*P* = 0.032, Student *t*-test), whereas the ratio of acetate to propionate (Acetate/Propionate) was significant higher (*P* = 0.035, Student *t*-test) in the HF cows when compared with those in the LF cows (Table 2). However, no significance (*P* > 0.05, Student *t*-test) was found for other aspects of the fermentation index or the FA (C > 8) composition of the rumen between the groups (Table 2).

**Table 2:** The rumen fermentation index of Holstein cows with highest milk fat percentage (HF) or lowest milk fat percentage (LF).

	**HF**	**LF**	**SEM**	***P* value^a^**
pH	6.27	6.28	0.04	0.870
Ammonia N (mg/dL)	15.44	14.59	1.22	0.740
L-Lactate (mmol/L)	0.20	0.18	0.02	0.596
TVFA (mmol/L)	116.16	112.99	3.18	0.631
Acetate (mmol/L)	72.35	68.45	1.93	0.325
Propionate (mmol/L)	23.99	25.59	0.99	0.436
Butyrate (mmol/L)	15.46	14.62	0.57	0.477
Isobutyrate (mmol/L)	1.00	0.95	0.03	0.378
Valerate (mmol/L)	1.67	1.65	0.07	0.926
Isovalerate (mmol/L)	1.68	1.73	0.08	0.777
Acetate (mol %)	62.39	60.60	0.57	0.117
Propionate (mol %)	20.60	22.54	0.47	0.032
Butyrate (mol %)	13.28	13.02	0.38	0.743
Acetate/Propionate	3.06	2.71	0.08	0.035

a P values were calculated by student t-test.

TVFA, total volatile fatty acids.

### 3.3. Milk fatty acid compositions

The compositions of milk FA (C > 8) in the two groups were shown in Table 3. Compared with the HF cows, the levels of C8:0, C10:0, C14:0, C15:0, *trans-*10, *cis-*12 C18:2, and *cis-*8,11,14 C20:3 were higher (*P* < 0.05, Student *t*-test), and the concentration of *cis-*9 C16:1 was lower (*P* = 0.003, Student *t*-test) in LF cows.

**Table 3:** Fatty acid (longer than 8C) compositions in milk and rumen fluid of Holstein cows with highest milk fat percentage (HF) or lowest milk fat percentage (LF) (g/100g of TFA).

	**Milk**				**Rumen fluid**			
	**HF**	**LF**	**SEM**	***P^a^***	**HF**	**LF**	**SEM**	***P^a^***
C8:0	0.76	0.97	0.05	0.039	0.66	0.76	0.08	0.531
C10:0	2.41	2.98	0.14	0.045	0.23	0.32	0.03	0.184
C11:0	0.28	0.35	0.02	0.083	Not detect			
C12:0	2.70	3.25	0.16	0.084	0.32	0.31	0.01	0.743
C13:0	0.12	0.17	0.01	0.069	0.13	0.12	0.01	0.765
C14:0	9.31	10.54	0.27	0.020	0.86	0.75	0.05	0.306
C14:1 *cis*-9	0.63	0.66	0.03	0.582	Not detect			
C15:0	0.76	0.92	0.04	0.036	0.52	0.60	0.02	0.075
C16:0	33.88	33.17	0.30	0.244	29.35	29.69	0.36	0.657
C16:1 *cis*-9	2.29	1.75	0.10	0.003	0.16	0.20	0.04	0.537
C17:0	0.59	0.60	0.01	0.826	0.20	0.20	0.01	0.950
C18:0	14.14	14.4	0.30	0.678	54.89	53.57	0.75	0.394
C18:1 *cis*-9	25.82	23.83	0.59	0.095	4.25	4.61	0.18	0.332
C18:1 *trans*-9	0.34	0.36	0.01	0.418	0.44	0.40	0.03	0.552
C18:1 *trans*-11	1.84	1.75	0.06	0.453	2.71	2.97	0.14	0.388
C18:2 *cis*-9,12	0.76	0.97	0.05	0.039	2.04	1.77	0.10	0.186
C18:2 *trans*-9,12	0.18	0.19	0.01	0.912	0.08	0.12	0.02	0.321
C18:2 *trans*-10, *cis*-12	0.01	0.03	<0.01	<0.001	0.04	0.04	<0.01	0.923
C18:3 *cis-*6,9,12	0.04	0.05	<0.01	0.528	0.56	0.56	0.01	0.983
C18:3 *cis*-9,12,15	0.36	0.33	0.01	0.168	0.86	1.26	0.13	0.110
C20:0	0.16	0.19	0.01	0.108	Not detect			
C20:1 *cis-*11	0.22	0.23	<0.01	0.365	0.36	0.36	0.03	0.997
C20:2 *cis*-11,14	0.02	0.02	<0.01	0.863	0.28	0.35	0.05	0.517
C20:3 *cis*-8,11,14	0.17	0.22	0.01	0.039	Not detect			
C22:1 *cis*-13	0.14	0.15	<0.01	0.148	0.10	0.10	0.01	0.964
C22:2 *cis*-13,16	0.04	0.10	0.02	0.074	Not detect			

TFA, total fatty acids.

^a^P values were calculated by student t-test.

### 3.4. Microbial diversity and taxonomic distribution of two groups

**Figure 1:**
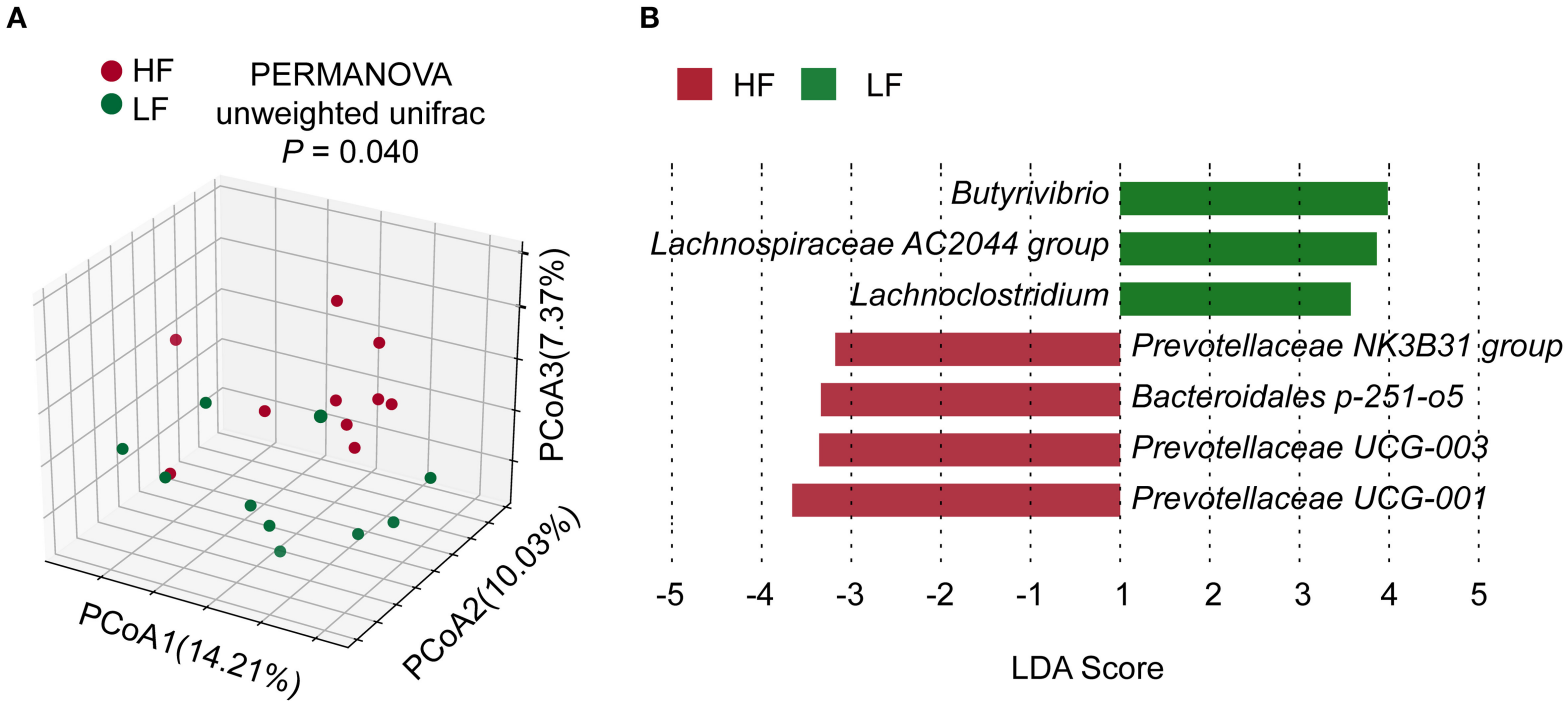
**(A)** Comparisons of bacterial community composition. **(B)** LEfSe identification of biomarker genera of the groups. HF, the top 10 cows with the highest milk fat percentage; LF, the last 10 cows with the lowest milk fat percentage; LDA, linear discriminant analysis.

The rarefaction curves of amplicon sequencing were shown in Supplementary Figure 2. The α-diversity of bacterial community showed no significant difference (*P* > 0.05) between the groups (Supplementary Table 3). However, β-diversity exhibited a significant difference (*P* = 0.040, PERMANOVA) between two groups (Figure 1A). There were no significant differences (*P* > 0.05, Wilcoxon rank-sum test) in taxa between the two groups in the phylum or genus levels (Supplementary Figure 3). Nevertheless, LEfSe analysis revealed that Prevotellaceae NK3B31 group, Bacteroidales p-251-o5, Prevotellaceae UCG-003, and Prevotellaceae UCG-001 contributed to the greatest extent (biomarkers in the LFfSe analysis) to the construction of the rumen bacterial community in the HF groups (Figure 1B). On the other hand, *Butyrivibrio*, Lachnospiraceae AC2044 group, and *Lachnoclostridium*, all of which belong to the family Lachnospiraceae, contributed the greatest extent (biomarkers in the LFfSe analysis) to the construction of the rumen bacterial community in the LF group.

### 3.5. Co-occurrence patterns of rumen microbes

**Figure 2:**
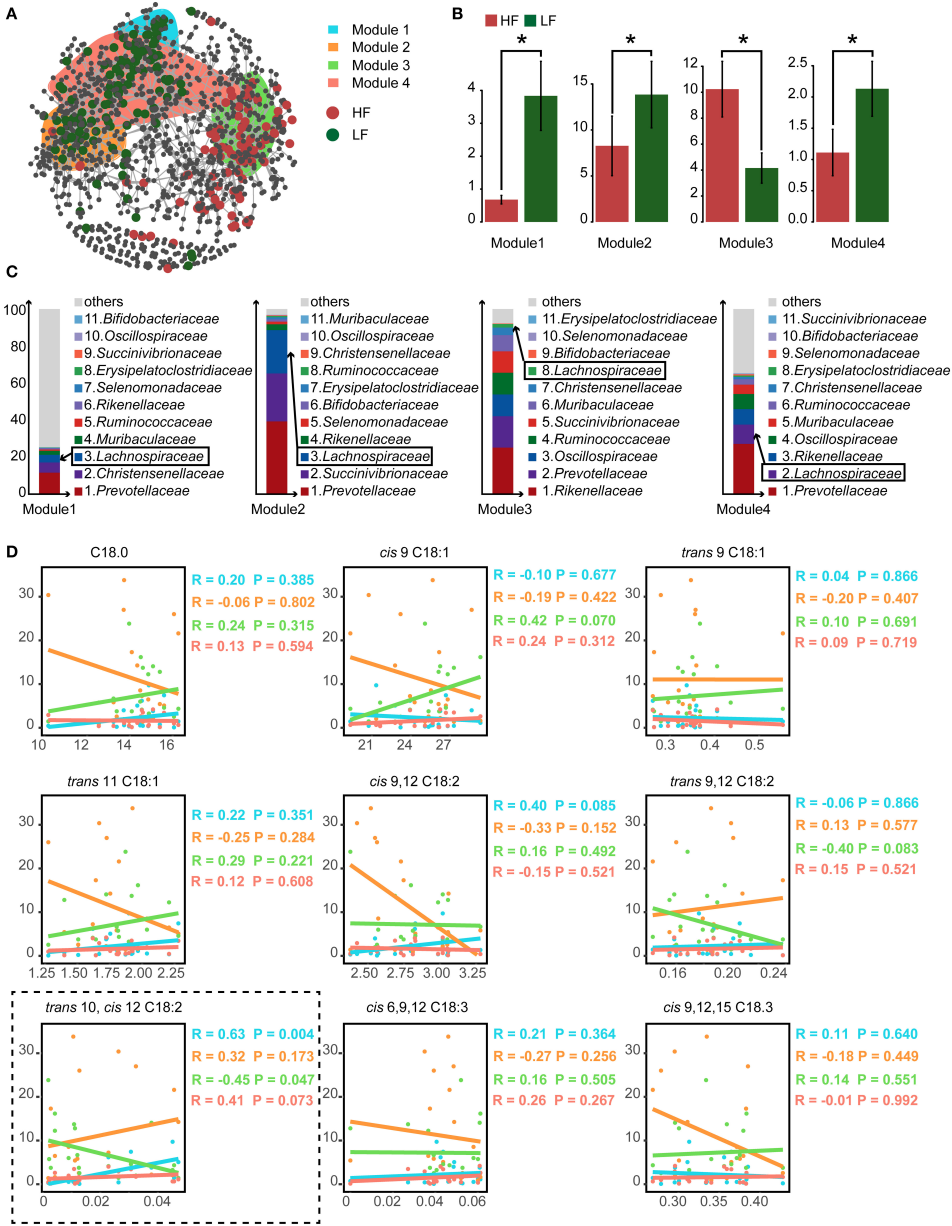
**(A)** The cooccurrence network of the significantly different amplicon sequence variants (ASVs). **(B)** The relative abundance of the predominant modules who contained the largest number of significantly different ASVs. **(C)** The compositions of bacterial family of the predominant modules. The ranking of family was based on the relative abundance. The ranking of Lachnospiraceae was marked with the box. **(D)** The spearman correlations between the relative abundance of predominant modules and the relative content of milk C18 fatty acids (FA). HF, the top 10 cows with the highest milk fat percentage; LF, the last 10 cows with the lowest milk fat percentage. *indicated *P* < 0.05 in the Wilcoxon rank-sum test.

To detect the ecological interactions of the ruminal bacteria, the co-occurrence network was constructed, with the ASVs having significantly different abundances falling into four modules (Figure 2A). Calculated as the total relative abundance of ASVs in modules, the relative abundances of modules 1, 2, and 4 were significant lower (*P* < 0.05), and the relative abundance of module 3 was significant higher (*P* < 0.05) in the HF cows when compared with those of the LF cows (Figure 2B). Furthermore, in the analysis of the compositions of these modules, the relative abundance of Lachnospiraceae was within the top 3 in modules 1, 2, and 4 at the family level (Figure 2C). In the analysis of correlations between the concentration of C18 FA and the relative abundances of the modules, a positive correlation of *trans-*10, *cis-*12 C18:2 with module 1 (Spearman's correlation coefficient *r*_*s*_ = 0.63, *P* = 0.004) and module 4 (*r*_*s*_ = 0.41, *P* = 0.073), and a negative correlation between *trans-*10, *cis-*12 C18:2 and module 3 (*r*_*s*_ = −0.45, *P* = 0.047) were detected (Figure 2D).

### 3.6. Variance in biohydrogenation

**Figure 3:**
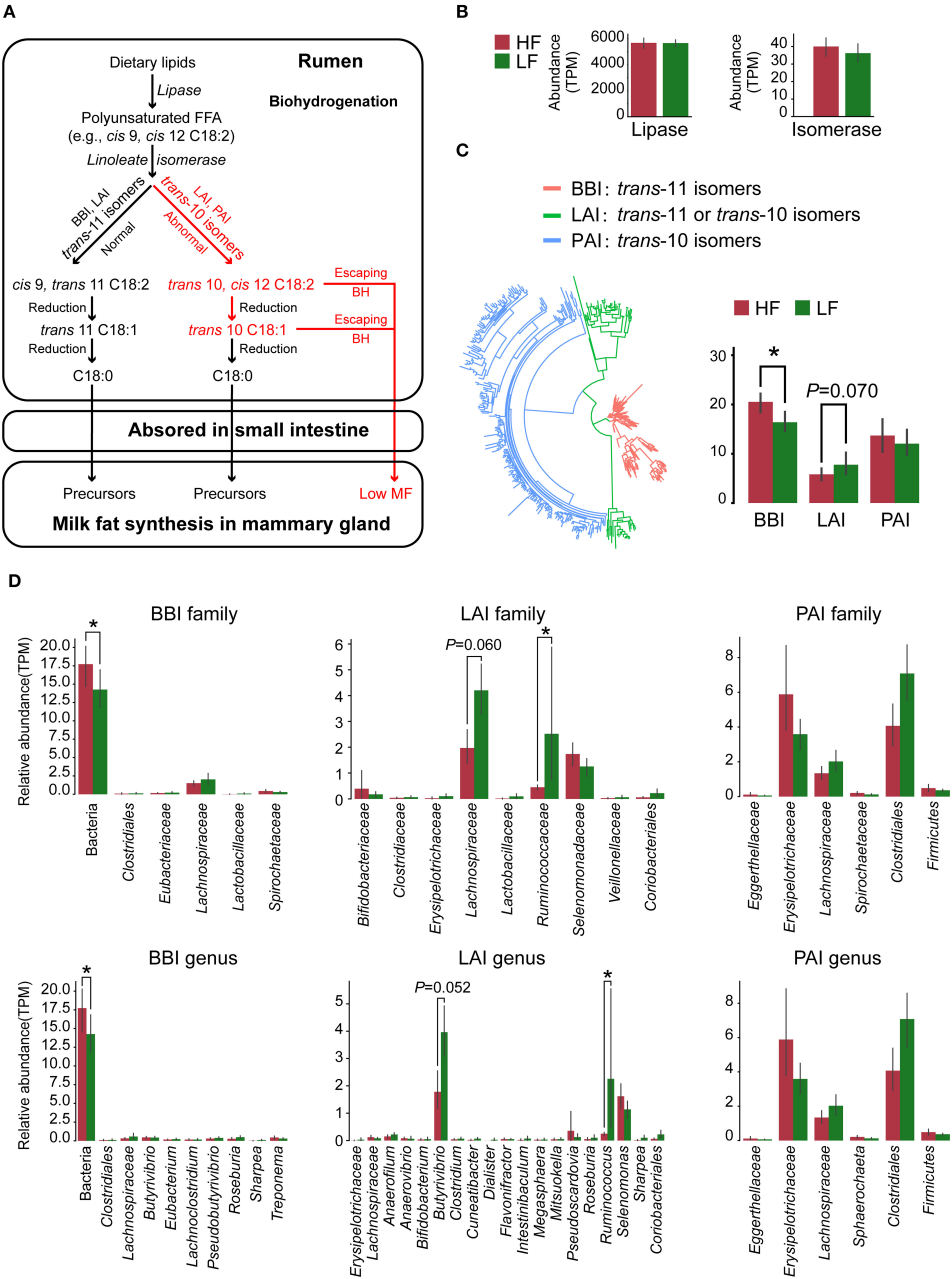
**(A)** Major biohydrogenation pathways in the production of C18 fatty acids in the rumen. **(B)** The relative abundance of lipase and isomerase genes identified in the metagenome data. **(C)** The classification and relative abundance of three types of isomerase genes identified in the metagenome data. **(D)** The taxonomic annotation and relative abundance of the isomerase genes. HF, the top 10 cows with the highest milk fat percentage; LF, the last 10 cows with the lowest milk fat percentage; BBI, *Bifidobacterium* linoleic acid (LA) isomerase; LAI, *Lactobacillus* LA isomerase; PAI *Propionibacterium* LA isomerase. *indicated that *P* < 0.05 in Wilcoxon rank-sum test.

Lipases and three types of LA/ALA isomerases have been detected in our metagenome sequencing data. The roles of these enzymes in the rumen BH process, based on previous reports (8), are shown in Figure 3A. Herein, the relative abundance of lipase showed no significant difference (*P* > 0.05) between the two groups (Figure 3B). However, the relative abundance of BBI was higher (*P* = 0.007, Wilcoxon rank-sum test), whereas the relative abundance of LAI tended to be lower (*P* = 0.070, Wilcoxon rank-sum test) in the HF cows when compared with the LF cows (Figure 3C). By analyzing the species with lipase genes, there was no significant difference (*P* > 0.05, Wilcoxon rank-sum test) of the predominant genera between the two groups (Supplementary Figure 4). In the analysis of the species with these isomerase genes, the relative abundance of *Butyrivibrio* with LAI tended to be lower (*P* = 0.052, Wilcoxon rank-sum test), and the relative abundance of *Ruminococcus* with LAI was lower (*P* = 0.037, Wilcoxon rank-sum test) in the HF cows, when compared with those in the LF cows (Figure 3D).

### 3.7. Correlations between MAGs and trans-10, cis-12 C18:2

**Figure 4:**
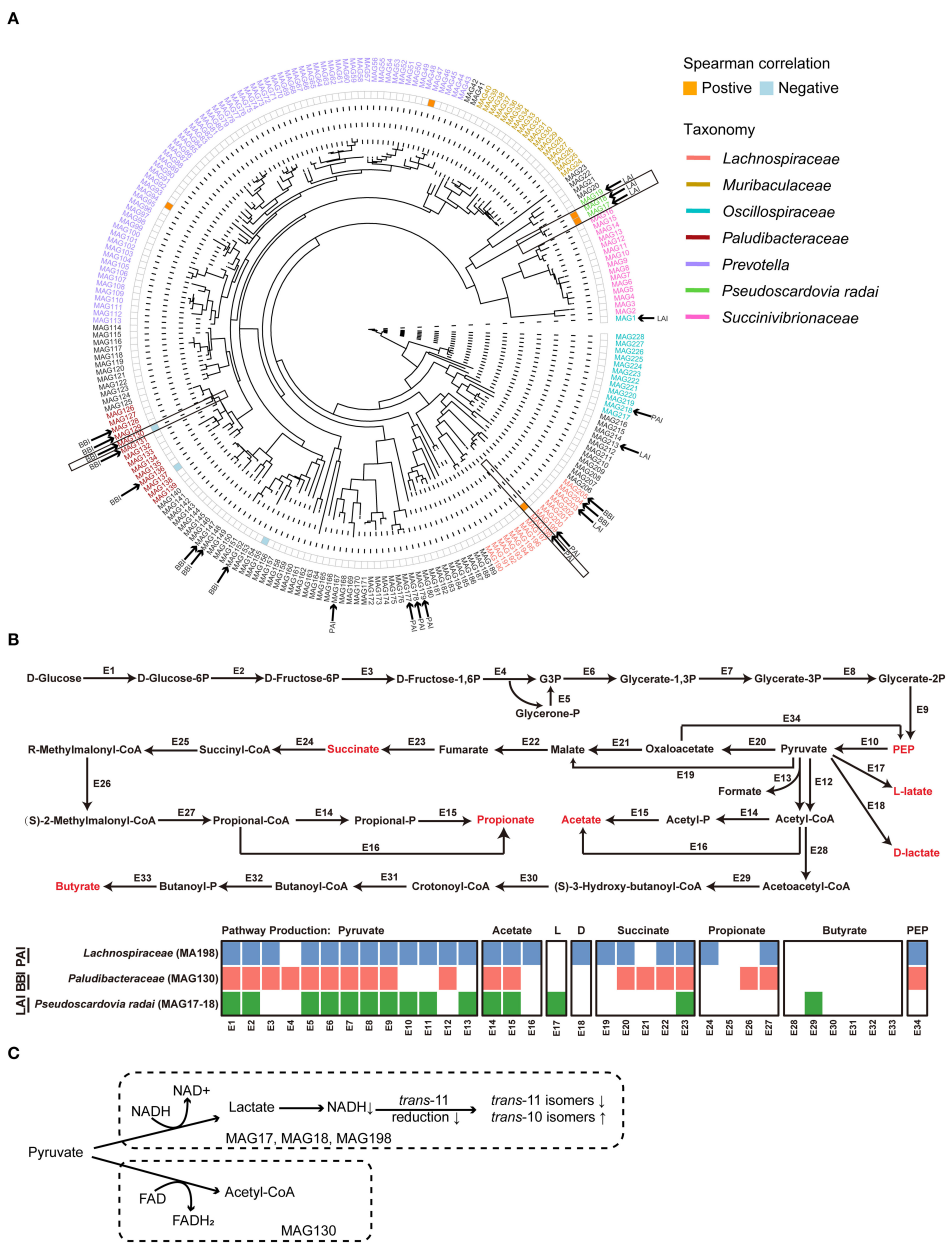
**(A)** The phylogenetic tree of 228 metagenome-assembled genomes (MAGs). The color orange and blue on the heatmap next to the tree represented significantly positive correlations and negative correlations between the relative abundance of MAGs and the relative content of milk *trans-*10*, cis-*12 C18:2, respectively. The MAGs who contained the linoleic acid (LA) isomerase were marked by the black arrow. The MAGs in black frame were selected to do the genomic analysis. **(B)** The glucose metabolism pathways of the selected MAGs. The detailed information of the enzyme genes was listed in Supplementary Table 4. **(C)** The hypothesis concerning the relationship of the major suppliers of reducing equivalents and the production of C18:2 isomers for the biohydrogenation bacteria in rumen. HF, the top 10 cows with the highest milk fat percentage; LF, the last 10 cows with the lowest milk fat percentage; BBI, *Bifidobacterium* linoleic acid (LA) isomerase; LAI, *Lactobacillus* LA isomerase; PAI *Propionibacterium* LA isomerase; NAD, nicotinamide adenine dinucleotide; FAD, flavin adenine dinucleotide.

As shown in the phylogeny tree, a total of 228 MAGs was obtained in this study (Figure 4A). Among them, a total of 23 MAGs carrying at least one kind of LA/ALA isomerase genes were identified. According to the taxonomy annotation results, 7 MAGs with PAI were assigned to Lachnospiraceae (two), Oscillospiraceae (one), and unclassified bacteria (four), 6 MAGs with LAI were *Pseudoscardovia radai* (three), Oscillospiraceae (one), Lachnospiraceae (one), and unclassified bacterium (one), and 10 MAGs with BBI were Paludibacteraceae (five), Lachnospiraceae (two), and unclassified bacteria (three).

Spearman's correlation analysis was performed between the relative abundance of MAGs with LA/ALA isomerase genes and the relative content of *trans*-10, *cis*-12 C18:2 in milk fat. The results showed that MAG with PAI (MAG198; Lachnospiraceae bacterium) and two MAGs with LAI (MAG17 & MAG18, *Pseudoscardovia radai*) were positively correlated with milk *trans-*10, *cis-*12 C18:2 (*r*_*s*_ > 0.4, *P* < 0.05, Figure 4A). On the other hand, one MAG with BBI (MAG130; Paludibacteraceae bacterium) was negatively correlated with milk *trans-*10, *cis-*12 C18:2 (*r*_*s*_ <−0.4, *P* < 0.05, Figure 4A). A reconstruction of the metabolic pathways of the above four MAGs (Supplementary Table 4) revealed that the three MAGs (MAG17, 18, and 198) positively correlated with milk *trans-*10, *cis-*12 C18:2 were able to produce lactate, whereas the MAG130 negatively correlated with milk *trans-*10, *cis-*12 C18:2 was not able to produced lactate (Figure 4B).

## 4. Discussion

The present study aimed to investigate the relationships between rumen microbiome and MFP, where two groups with no significant differences in their body weight, DIM, parity, milk yield, milk protein, and lactose percentage were selected for experiments. Results showed a significant difference in MFP between two groups for a total of 5 weeks, indicating that the disparity was not attributed to a random error of sampling. According to our results, the rumen pH, rumen VFA concentrations, and rumen FAs concentrations exhibited no significant differences between the groups. However, the rumen acetate/propionate, being an important index reflecting microbial community structure, and the β-diversity of ASVs showed the minor but significant differences between the groups (44). Accordingly, we inferred that a minor difference involving a small number of microbes existed between the composition of the rumen microbiome in the two groups. Notably, the concentration of milk *trans-*10, *cis-*12 C18:2, being a potent inhibitor of milk fat synthesis (6), significantly increased in the LF cows compared with the HF cows. Because of the absence of Δ12 desaturase in animal tissue, milk *trans-*10, *cis-*12 C18:2 should be derived from the BH process of ruminal microbes (45), which suggested that the rumen BH pathways or levels would be different between the groups. In the present study, the inconsistency of rumen *trans-*10, *cis-*12 C18:2 might have been induced by the difference in its ruminal escape or in the metabolism efficiency between the groups. Overall, these results implied that the variance in the microbial population undergoing BH within the rumen microbiome contribute to the variance of MFP.

As shown in Figure 3A, two key enzymes, the lipases for lipolysis and the isomerases for LA/ALA isomerization, were involved in BH process (8). There was no difference in the relative abundance of lipases and their taxonomy, which indicated the variance of rumen BH pathways might be in LA/ALA isomerization between two groups. According to the available studies (6, 8), the *trans*-10 BH pathway that produces *trans-*10, *cis-*12 C18:2 intermediates and the *tran*-11 BH pathway that produces *cis-*9, *trans-*11 C18:2 intermediates are two major pathways for LA/ALA isomerization in the rumen (Figure 3A). However, knowledge concerning the species involved in these pathways is limited. A recent study has found that the enrichment of the *trans*-10 BH pathway is related to an increase in the relative abundance of Lachnospiraceae (6). In our study, *Butyrivibrio*, Lachnospiraceae AC2044 group and *Lachnoclostridium*, all of which belong to the family Lachnospiraceae, have been found to be the biomarker genera for the LF group, suggesting a dominant role of the *trans*-10 BH pathway in the LF group. Our co-occurrence network analysis has also revealed that Lachnospiraceae is the predominant family in the modules 1, 2 and 4 who were significantly enriched in the LF group. Furthermore, modules 1 and 4 are positively correlated with *trans-*10, *cis-*12 C18:2. Bauman and Griinari (7) have proposed that the production of *trans-*10, *cis-*12 C18:2, rather than *cis-*9, *trans-*11 C18:2, from LA isomerization, is one reason for the occurrence of low milk fat syndrome in dairy cows. Taking these data together, we inferred that the dominance of bacteria with *trans*-10 BH pathway in rumen microbiome, especially Lachnospiraceae, leads to an increased production efficiency of *trans-*10, *cis-*12 C18:2, contributing to the decrease of MFP in the LF group.

So far, three types of LA/ALA isomerase with different isomerization production and derived species have been reported: (1) BBI, which was identified from *Bifidobacterium* and was found to produce *trans*-11 isomers (32), (2) PAI that was identified from *Propionibacterium* and was found to produce *trans*-10 isomers (46), and (3) LAI that was identified from *Lactobacillus* and was found to produce *trans*-11 or *trans*-10 isomers (47). Here, the above three isomerase genes have been detected in our results, where we have found a significantly increased relative abundance of BBI and a decreased trend regarding the relative abundance of LAI in the HF cows compared with the LF cows (Figure 3C). Our results further support our previous hypothesis at the gene level that changes in the BH microbial populations of the rumen leads to changes in the production efficiency of *trans-*10, *cis-*12 C18:2 in the LF group.

For a greater understanding of the possible reasons for the shaping of the BH microbial populations, we reconstructed the glucose metabolism pathways of the MAGs that were highly correlated to the concentration of milk *trans-*10, *cis-*12 C18:2 (Figure 4B). As a result, in the metabolism pathways of three MAGs (MAG17, 18 and 198) that were positively correlated to *trans-*10, *cis-*12 C18:2, the reduction of pyruvate was found to be associated with the oxidation of reduced nicotinamide adenine dinucleotide (NADH). On the other hand, in the metabolism pathways of MAG130 that was negatively correlated to *trans*-10, *cis*-12 C18:2, the reduction of pyruvate was associated with the oxidation of reduced flavin adenine dinucleotide (FADH_2_). Previous studies showed that reduction of *cis-9, trans-11* C18:2 required the oxidation of NADH, and the production of *trans*-10, *cis*-12 C18:2 required the oxidation of FAD H_2_ (48, 49). Hence, we infer that the usage of NADH or FADH_2_ as the major supplier of reducing equivalents affected the choice of *trans-11* or *trans*-10 BH pathway, and thereafter, the production of *cis-9, trans-11* C18:2 or *trans-*10, *cis-*12 C18:2 for the BH bacteria in the rumen (Figure 4C). However, the hypothesis needs further investigation.

## 5. Conclusion

The increased relative abundance of microbial population with the *trans*-10 BH pathway within the rumen microbiome, especially Lachnospiraceae, contributes to the decrease of MFP *via* the increase of rumen *trans-*10, *cis-*12 C18:2 production. Our study provides a new perspective for the development of measures for improving the milking performance of dairy cows.

## Data availability statement

The datasets presented in this study can be found in online repositories. The names of the repository/repositories and accession number(s) can be found below: https://www.ncbi.nlm.nih.gov/sra/PRJNA883555, https://www.ncbi.nlm.nih.gov/sra/PRJNA883576, https://doi.org/10.6084/m9.figshare.21211136.

## Ethics statement

The animal study was reviewed and approved by Regulations for the Care and Use of Animals (Nanjing Agricultural University, 2018).

## Author contributions

LZ and JZ conducted animal experiments. The statistical analysis and manuscript were performed by LZ and HS. The experiment design and funding were from SM. All authors have read the manuscript.
