# Supplementary material for: Variety of rumen microbial populations involved in biohydrogenation related to individual milk fat percentage of dairy cows
Source: Front Vet Sci. 2023 Mar 2;10:1106834. doi: 10.3389/fvets.2023.1106834 (PMC10019597; doi:10.3389/fvets.2023.1106834)
Supplement: Supplementary file 1 [file Data_Sheet_1.docx]

1 Supplementary Figures and Tables

1.1 Supplementary Figures


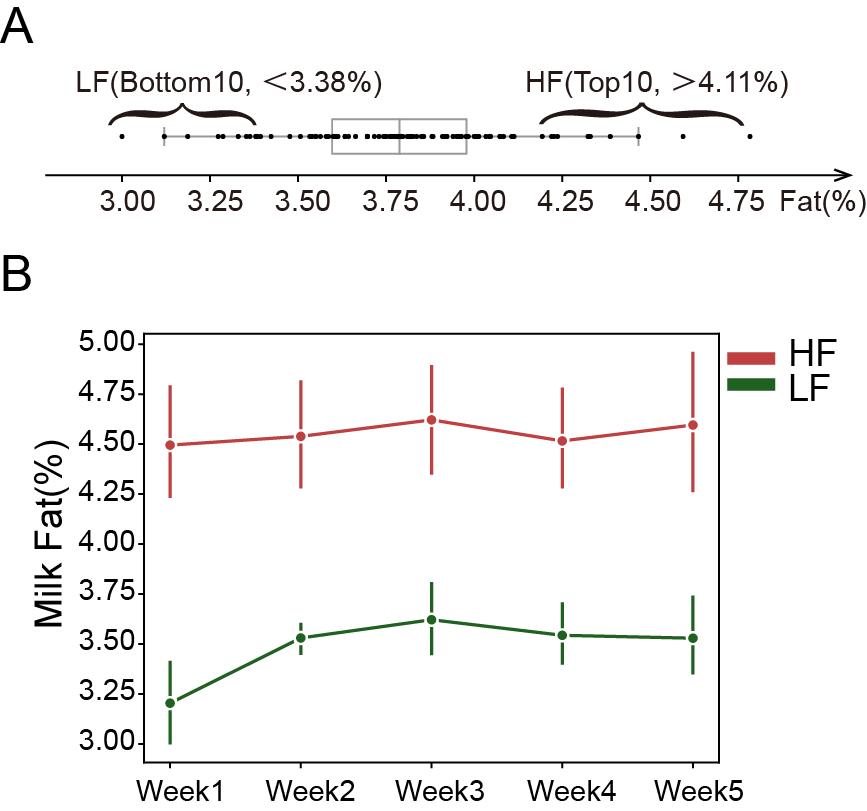


Figure S1. (A) The diagram about how to select 20 cows from the herd. The bottom 10 cows with milk fat percentage (MFP) < 3.38% were regarded as LF cows, and top 10 cows whose MFP > 4.11% were regarded as HF cows. (B) MFP of two groups in the next five weeks after sampling remained disparity.


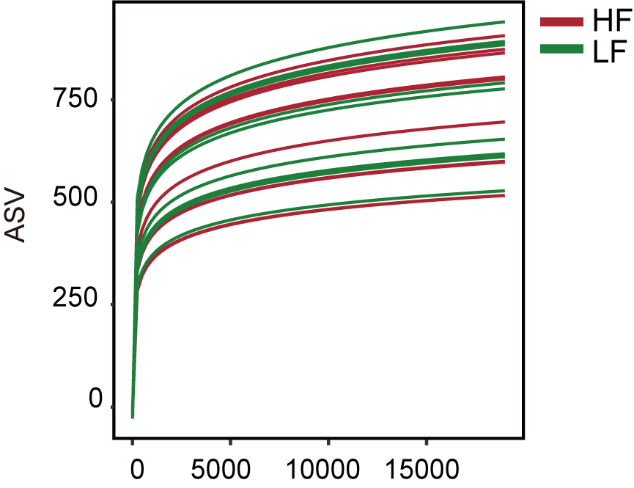


Figure S2. The rarefaction curves of each sample. The plot indicated that the sequencing depth met experimental requirements. HF, the top 10 cows with the highest milk fat percentage; LF, the last 10 cows with the lowest milk fat percentage.


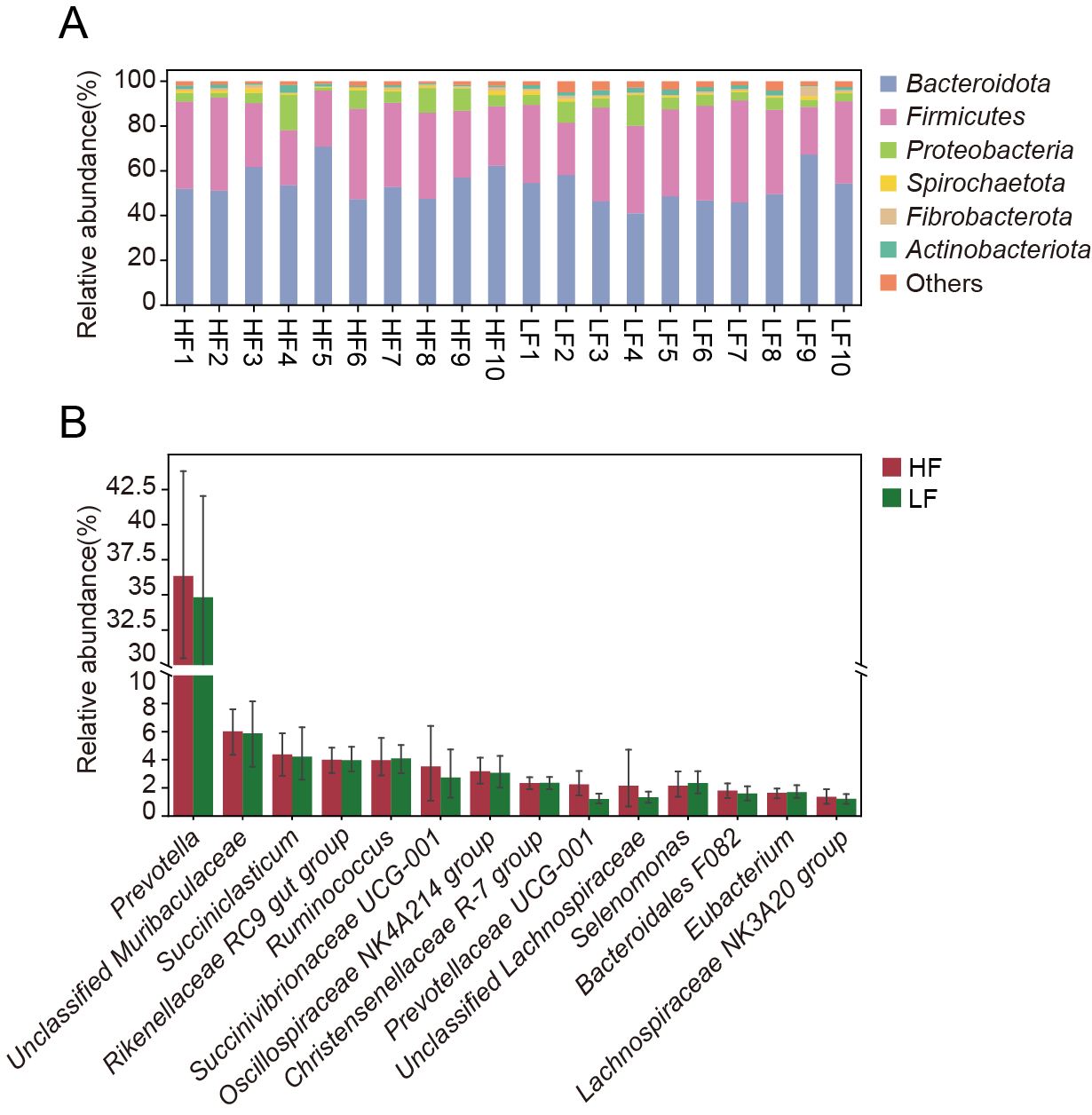


Figure S3. The taxonomic distribution of two groups at the (A) phylum and (B) genus level. HF, the top 10 cows with the highest milk fat percentage; LF, the last 10 cows with the lowest milk fat percentage.


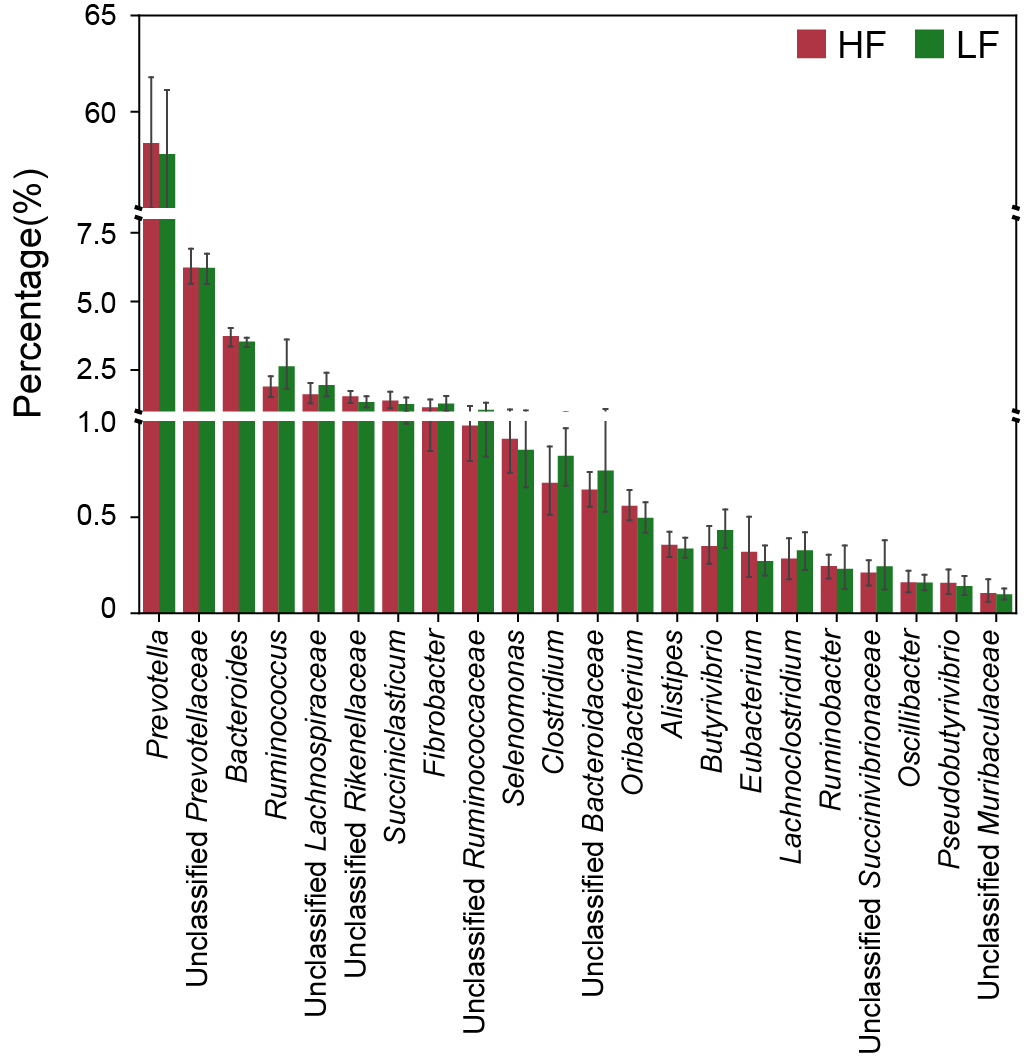


Figure S4. The taxonomic annotations of those sequences mapped to lipase. HF, the top 10 cows with the highest milk fat percentage; LF, the last 10 cows with the lowest milk fat percentage.

1.2 Supplementary Tables

Table S1. Ingredients and chemical composition of the diet

| Feed ingredients | Percentage |
| --- | --- |
| Maize | 26.87 |
| Soybean meal | 11.52 |
| Extruded soybean | 3.43 |
| Soy hull | 3.43 |
| Whole Cottonseed | 6.86 |
| Alfalfa hay | 6.17 |
| Oat hay | 5.14 |
| Alfalfa silage | 3.50 |
| Maize silage | 25.70 |
| Molasses | 3.12 |
| Yeast Culture | 0.12 |
| Lysine | 1.17 |
| Methionine | 0.04 |
| Palm fat powder | 0.12 |
| Urea | 0.31 |
| Vitamin and mineral mix^1^ | 2.50 |
| Chemical composition^2^ |  |
| CP, % | 17.46 |
| NDF, % | 30.89 |
| ADF, % | 18.98 |
| Ether extract, % | 5.43 |
| Ash, % | 9.3 |

^1^ Premix contained the following ingredients: vitamin A, 180KIU/kg; vitamin D3, 45KIU/kg; vitamin E, 1400KIU/kg; Fe, 170mg/kg; Cu, 360mg/kg; Zn, 680mg/kg; Mn, 910mg/kg; Co, 4mg/kg; I, 20mg/kg; Se, 6mg/kg

^2^ dry matter basis

Table S2. The lactation performances of 92 dairy cows (Mean ± SD).

|  | 92 cows |
| --- | --- |
| Days in milking | 56.82 ± 14.83 |
| Weight(kg) | 631.35 ± 69.70 |
| Milk fat(%) | 3.79 ± 0.47 |
| Milk protein(%) | 2.94 ± 0.20 |
| Milk Lactose(%) | 5.26 ± 0.13 |
| Milk yield(kg) | 36.90 ± 7.12 |

Table S3. Alpha diversity of rumen ASVs of Holstein cows with highest milk fat percentage (HF) or lowest milk fat percentage (LF).

|  | HF | LF | SEM | *P* value^1^ |
| --- | --- | --- | --- | --- |
| Chao1 | 743.56 | 794.33 | 37.32 | 0.511 |
| Shannon | 8.16 | 8.15 | 0.15 | 0.955 |
| Simpson | 0.99 | 0.98 | 0.01 | 0.625 |

^1^ *P* values were calculated by Wilcoxon rank-sum test

Table S4. The genes of selected MAGs on glucose metabolism

| Pathway production | Enzyme ID | EC NO | MAG198 | MAG130 | MAG17-18 |
| --- | --- | --- | --- | --- | --- |
| Pyruvate | E1 | 2.7.1.2 | K25026 | K25026 | K25026 |
|  | E2 | 5.3.1.9 | K01810 | K01810 | K01810 |
|  | E3 | 2.7.1.11 | K00850\|K21071 | K00850 |  |
|  | E4 | 2.7.1.90 |  | K00895 |  |
|  | E5 | 4.1.2.13 | K01624 | K01624 | K01624 |
|  | E6 | 5.3.1.1 | K01803 | K01803 | K01803 |
|  | E7 | 1.2.1.12 | K00134 | K00134 | K00134 |
|  | E8 | 2.7.2.3 | K00927 | K00927 | K00927 |
|  | E9 | 5.4.2.11;5.4.2.12 | K15634;K15633 | K15633 | K01834 |
|  | E10 | 4.2.1.11 | K01689 |  | K01689 |
|  | E11 | 2.7.1.40 | K00873 |  | K00873 |
|  | E12 | 1.2.7.1;1.2.7.11 | K03737;K00174\|K00175 | K03737;K00174\|K00175 |  |
|  | E13 | 2.3.1.54 | K00656 |  | K00656 |
| Acetate | E14 | 2.3.1.8 | K00625 | K00625 | K13788 |
|  | E15 | 2.7.2.1;3.6.1.7 | K00925;K01512 | K00925 | K00925 |
|  | E16 | 3.1.2.1;6.2.1.1 | K01067;K01895 |  |  |
| L-lactate | E17 | 1.1.1.27 |  |  | K00016 |
| D-lactate | E18 | 1.1.1.28 | K03778 |  |  |
| Succinate | E19 | 1.1.1.38 | K00027 |  |  |
|  | E20 | 6.4.1.1 | K01960 | K01960 |  |
|  | E21 | 1.1.1.37 |  | K00024 |  |
|  | E22 | 4.2.1.2 | K01679 | K01676 |  |
|  | E23 | 1.3.5.1 | K18560 | K00239\|K00240 | K00239\|K00240 |
| Propionate | E24 | 6.2.1.1 | K01895 |  |  |
|  | E25 | 5.4.99.2 |  |  |  |
|  | E26 | 5.1.99.1 |  | K05606 |  |
|  | E27 | K23351\|K23352 | K23351\|K23352 | K23351 |  |
| Butyrate | E28 | 2.3.1.9 |  |  |  |
|  | E29 | 1.1.1.157 |  |  | K00074 |
|  | E30 | 4.2.1.17 |  |  |  |
|  | E31 | 1.3.8.1 |  |  |  |
|  | E32 | 2.3.1.19 |  |  |  |
|  | E33 | 2.7.2.7 |  |  |  |
| Phosphoenolpyruvate | E34 | 4.1.1.49;4.1.1.32 | K01610 | K01610 |  |
